# Association of frailty-risk indicators with cardiovascular diseases: evidence from the CHARLS

**DOI:** 10.1186/s12872-026-06213-w

**Published:** 2026-07-08

**Authors:** Fangfang Zhuo, Zhao’ xing Cao, Zhipeng Pu, Run’ ze Huang, Zhang’ rong Chen

**Affiliations:** 1 Guizhou Medical University, Guiyang, Guizhou 550004 China; 2 Department of Cardiovascular Medicine, The Affiliated Hospital of Guizhou Medical University, No. 28, Guiyi Street, Yunyan District, Guiyang City, Guizhou Province 550004 China; 3 The Key Laboratory of Myocardial Remodeling Research, The Affiliated Hospital of Guizhou Medical University, Guiyang, Guizhou 550004 China; 4 Center for Transforming Medical Research, Guizhou Medical University, Guiyang, Guizhou 550025 China

**Keywords:** China Health and Retirement Longitudinal Study (CHARLS), Frailty risk factors, Handgrip strength/weight, Cystatin C, Cardiovascular diseases

## Abstract

Cardiovascular diseases (CVDs) impose a heavy global disease burden, and China faces increasing healthcare costs and socioeconomic losses. Frailty is an age-related clinical state characterized by reduced physiological reserve and multisystem dysfunction. Because a complete Fried frailty phenotype could not be reconstructed from the available CHARLS variables, this study used “frailty risk factors” to refer to available physical performance and biomarker indicators related to frailty or high frailty risk, including handgrip strength (HGS)-derived variables and cystatin C (Cys C). This study explored the association between these indicators and CVDs.

**Methods** Data from the 2015 China Health and Retirement Longitudinal Study (CHARLS) were analyzed. Participants were grouped by prevalent CVDs, defined as self-reported physician-diagnosed hypertension, heart disease, or stroke. LASSO regression followed by simple and multiple logistic regression was used to screen variables. The dataset was randomly split 6:2:2 into training, testing, and validation sets to develop six models (LR, XGBoost, LightGBM, SVM, RF, and DT), and model performance was evaluated by discrimination, calibration, decision curve analysis, and SHAP interpretability analyses.

**Results** A total of 10,657 participants were included (76% female), of whom 2,650 had CVDs (84% female). The XGBoost model showed the best overall performance. SHAP analysis identified age, HbA1c, UA, and Cys C as variables increasing the predicted risk of CVDs, whereas higher left HGS/weight ratio and lower blood pressure values were associated with lower predicted risk.

**Conclusion** These findings suggest that frailty risk factors, especially HGS/weight ratio and Cys C, may provide additional information for CVD risk stratification beyond conventional factors. The results support incorporating simple physical performance assessment and frailty-risk evaluation into CVD prevention strategies.

**Supplementary Information:**

The online version contains supplementary material available at https://doi.org/10.1186/s12872-026-06213-w.

## Background

Cardiovascular diseases (CVDs) remain the leading cause of global morbidity and mortality and represent a major public health challenge in China. Population aging, longer life expectancy, and lifestyle changes have increased the absolute burden of CVDs, even though prevention and treatment strategies have continued to improve [1–6]. Traditional risk factors such as hypertension, dyslipidemia, smoking, alcohol consumption, diabetes and obesity are well established [7–12], but they do not fully capture age-related vulnerability and functional decline.

Frailty is defined as an age-related clinical state characterized by reduced physiological reserve, multisystem dysfunction, and increased vulnerability to external stressors. It is potentially reversible and differs from normal aging and disability [13]. The Fried phenotype is a widely used frailty framework based on five items: unintentional weight loss, exhaustion, low grip strength, slow gait, and low physical activity [14]. In the present study, the full Fried frailty phenotype could not be reconstructed from the available CHARLS variables. Therefore, the term “frailty risk factors” was used to denote available variables that are biologically or clinically associated with frailty or high frailty risk, rather than a confirmed diagnosis of frailty.A large number of clinical studies have confirmed that frailty is closely related to cardiovascular diseases including stroke, which further highlights the importance of early identification of frailty and its reasonable management throughout the whole process [15–17].Therefore, frailty, especially among the elderly, has been described as “without question, one of the most serious public health challenges we will face in this coming century” [18].

Among these variables, HGS reflects muscle strength and physical reserve, while Cys C is a renal-function-related biomarker that has also been associated with inflammation, physical decline, and frailty progression [19–27]. Evidence linking frailty and CVDs is increasing, but population-based data from China remain limited. Therefore, this study used the 2015 CHARLS dataset to evaluate the association between frailty risk factors and CVDs and to develop interpretable machine-learning models for CVD risk stratification.

## Materials and methods

### Data

CHARLS is a nationally representative longitudinal study conducted on Chinese middle-aged and elderly people aged 45 and above and their spouses. The study aims to comprehensively assess the social, economic, and health status of community residents. The CHARLS database contains extremely rich content, covering various aspects such as the basic personal information, family structure, health status, lifestyle, economic situation, work and retirement status, medical insurance, and utilization of medical services of the middle-aged and elderly population. It can accurately reflect the health status of the middle-aged and elderly groups in China [28].

The CHARLS national baseline survey was carried out in 2011, using a multistage sampling method with probability proportional to size. Its samples widely cover 28 provinces, 150 counties, and 450 villages, involving approximately 10,000 households and more than 17,000 people. This is a continuous survey, and participants are examined every 2–3 years. During data collection, participants received face-to-face interviews at home through computer-assisted personal interview technology. The CHARLS dataset can be downloaded from its official homepage (http://charls.pku.edu.cn/en). In this study, the 2015 survey data were selected. According to strict inclusion and exclusion criteria, and after processing missing values, 10,657 participants were finally included. Because this analysis used cross-sectional 2015 data, the model was intended to identify CVD-related risk patterns and classify high-risk participants within this dataset, not to predict contemporary or future CVD events in 2026.

All CHARLS data have obtained ethical approval from the Institutional Review Board of Peking University. The CHARLS project applied to the Biomedical Ethics Committee of Peking University and was granted permission for use (approval number: IRB00001052-11015).

### Observation indicators

The observation indicators were categorized as follows. General characteristics included age (years), gender, height, weight, waist circumference, knee height, smoking status, drinking status, white blood cell (WBC) count, hemoglobin (HGB), hematocrit (HCT), mean corpuscular volume (MCV), and platelet (PLT) count. CVD-related variables included systolic blood pressure (SBP), diastolic blood pressure (DBP), triglyceride (TG), high-density lipoprotein (HDL), low-density lipoprotein (LDL), cholesterol (Cho), glucose (Glu), glycated hemoglobin (HbA1c), uric acid (UA), creatinine (Cr), blood urea nitrogen (BUN), and C-reactive protein (CRP). Frailty risk factors included left HGS, right HGS, left/right HGS-to-weight ratio, sit-to-stand time, walking time or gait speed, and Cys C. Gender, smoking status, drinking status, and CVD occurrence were treated as categorical variables; the remaining indicators were treated as continuous variables.

Height, weight, waist circumference, knee height, blood pressure, HGS, sit-to-stand time and walking time were obtained from standardized CHARLS physical measurements. Blood biomarkers were derived from CHARLS laboratory examinations. Units followed the original CHARLS data dictionary, including mmHg for blood pressure, kg for HGS, seconds for performance tests, percentage for HbA1c, and mg/L for Cys C and CRP; other biochemical variables were analyzed in their original laboratory units. Because the present study focused on statistical associations and model development, clinical reference ranges were not used for variable recoding unless explicitly stated.

Prevalent CVDs were defined according to self-reported physician diagnosis in CHARLS. Participants were classified as having CVDs if they reported hypertension, heart disease (including coronary heart disease, myocardial infarction, heart failure or other organic cardiac disorders), or stroke. HGS (kg) of the left and right hands was measured twice using an electronic dynamometer, and the maximum value for each hand was retained. The five-repetition sit-to-stand test (s) recorded the time required to complete five stand-sit cycles without hand support. Walking time (s) was measured over a 2.5-m course at the usual pace, and gait speed (m/s) was calculated as distance divided by time. Low physical performance was defined as HGS < 27 kg in men or < 17 kg in women, five-time sit-to-stand time ≥ 12 s, or gait speed < 0.6 m/s according to Fried frailty criteria and AWGS-related criteria.

### Machine learning algorithms

Logistic Regression (LR) is a generalized linear statistical method for binary classification. It applies the sigmoid function to map the linear combination of independent variables into the range [0,1] and outputs the estimated probability of event occurrence, thereby allowing classification according to a predefined probability threshold [29].

Extreme Gradient Boosting(XGBoost) improves gradient boosting by iteratively training residual-based CART trees with pruning and regularization to avoid overfitting. It efficiently captures nonlinear patterns for high-dimensional classification and regression [30].

Proposed by Microsoft in 2017, Light Gradient Boosting Machine(LightGBM) builds ensemble trees via residual fitting. With histogram optimization and leaf-wise splitting, it outperforms XGBoost in memory cost and efficiency for high-dimensional big data [31].

Support Vector Machine (SVM) is a statistical learning-based classifier aiming to find the optimal hyperplane with maximum margin. It uses hard margin for linearly separable data and kernel functions to map nonlinear data into high-dimensional space. SVM performs well on high-dimensional small-sample data with strong generalization in binary classification [32].

Random forest (RF) is an ensemble learning algorithm rooted in decision trees. It generates numerous trees via bootstrap resampling of raw data and random feature selection for node splitting. Final predictions are aggregated across all constituent trees; independent training of individual trees also accelerates model fitting [33].

Decision tree (DT) serves as a decision-analytic technique built on event occurrence probabilities. It constructs tree structures to calculate relevant probabilities where the expected net present value reaches no less than zero. As an intuitive graphical tool for probabilistic analysis, DT features low computational complexity, interpretable outputs and good tolerance to missing intermediate data [34].

### Statistical analysis

In this study, all data were processed and analyzed using R 4.4.1. For metric data, normal–distributed data were presented as mean ± SD, while non–normal data were described by median (IQR). Depending on data distribution, t–test or Mann–Whitney U test was used for between–group comparisons. Categorical variables were shown as frequencies, and chi–square test was applied for group–difference analysis.

Pearson correlation analysis was applied only to continuous variables to evaluate pairwise linear associations. Binary variables, including gender, smoking status, drinking status, and CVD occurrence, were not included in the Pearson correlation heatmap. The correlation coefficient r was used to assess correlation strength: an absolute r value < 0.3 indicated weak correlation, 0.3–0.5 indicated moderate correlation, and > 0.5 indicated strong correlation. *P* < 0.05 was considered statistically significant. Highly correlated variables were considered when selecting variables for subsequent multiple logistic regression and machine-learning model construction.

Due to the large number of baseline variables, LASSO regression with tenfold cross-validation was used for preliminary feature selection. The value of lambda.min, which yielded the minimum cross-validated binomial deviance, was selected as the main feature-selection criterion to retain potential predictors for subsequent exploratory analysis.

Variables filtered by LASSO regression underwent simple and multiple logistic regression. Original data were split into training, testing, and validation sets at a 6:2:2 ratio. Variables significant in multiple logistic regression were included in the training set for building machine–learning models. Model performance was internally validated in testing and validation sets. The best—performing model was selected by evaluating performance metrics.

Finally, SHAP analysis explored the impact patterns and degrees of variables in the best model on CVDs occurrence. *P* < 0.05 was set as the statistical significance threshold in all analyses.

## Results

### Baseline characteristics

**Fig. 1:**
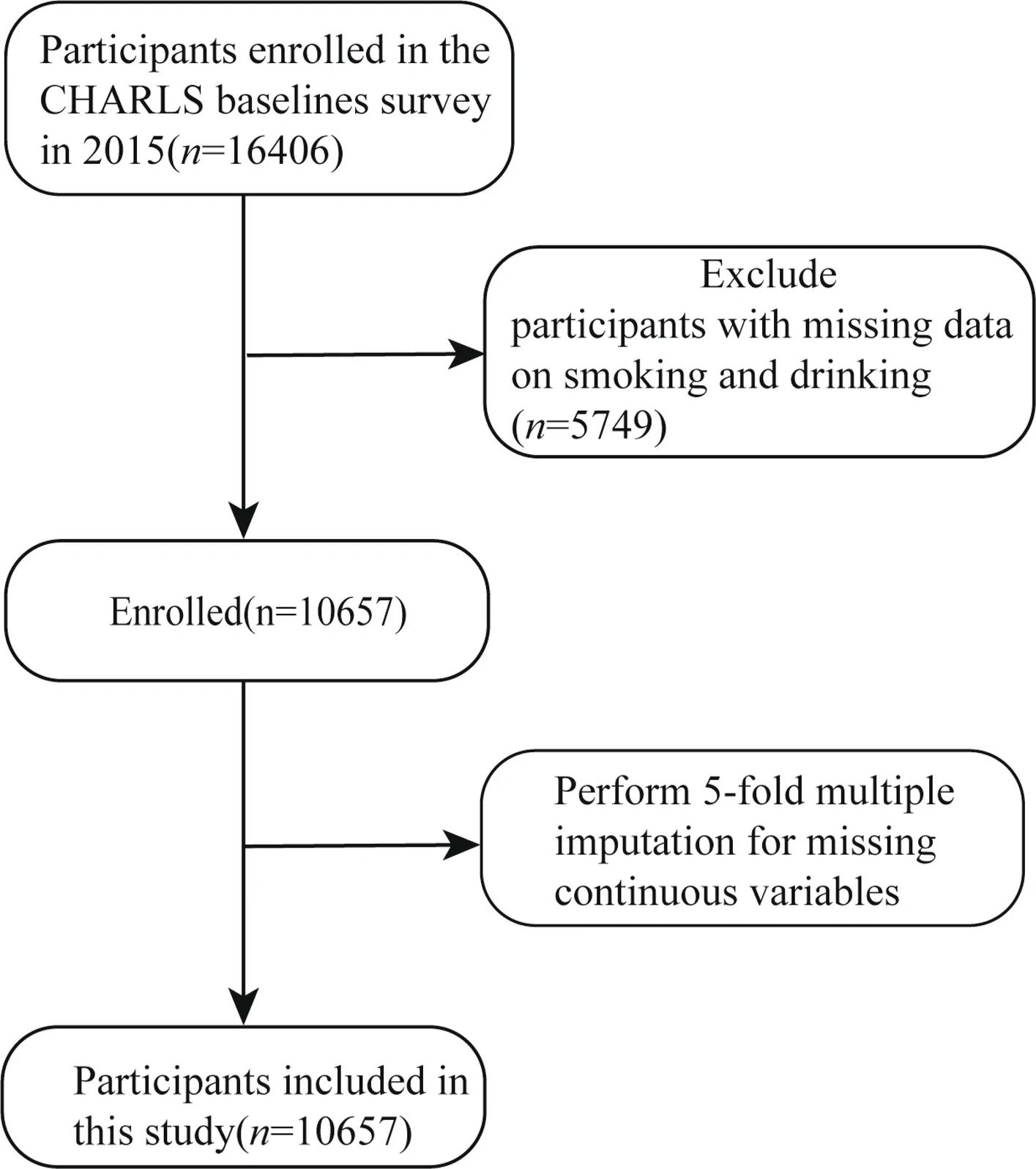
Flowchart of participant selection. A total of 16,406 cases from the 2015 CHARLS dataset were initially enrolled. Cases with missing smoking or drinking history were excluded, and missing continuous variables were supplemented via five-fold multiple imputation [29]. Finally, 10,657 cases were included in the analysis

A total of 10,657 participants were finally included (76% were females), among whom 2,650 had CVDs (84% were females). Except for walking time (*P* = 0.84), HGB (*P* = 0.15), MCV (*P* = 0.56), and PLT (*P* = 0.13), all other indicators were statistically significant (*P* < 0.001) (Table 1 and Fig. 1).

**Table 1:** Baseline data of patients with CVDs’ and non-CVDs’patients

Variables	Overall	Non-CVDs	CVDs	*p*
	N = 10,657	N = 8,007	N = 2,650	
Age(year)	58.00 (50.00–66.00)	55.00 (48.00–64.00)	63.00 (57.00–70.00)	< 0.001
Gender, n (%)				< 0.001
Male	2,520 (24)	2,106 (26)	414 (16)	
Female	8,137 (76)	5,901 (74)	2,236 (84)	
SBP(mmHg)	124.00 (111.50–138.00)	120.00 (109.50–133.00)	135.50 (122.00–149.50)	< 0.001
DBP(mmHg)	73.50 (66.50–81.50)	72.50 (65.50–80.00)	77.50 (70.00–86.00)	< 0.001
Height(cm)	155.10 (150.20–160.80)	155.60 (150.60–161.30)	154.00 (149.40–159.00)	< 0.001
Weight(kg)	58.20 (51.40–65.90)	57.70 (51.00–65.20)	59.80 (52.20–68.00)	< 0.001
BMI(kg/m2)	24.03 (21.70–26.58)	23.68 (21.48–26.18)	25.14 (22.66–27.64)	< 0.001
Waist circumference(cm)	86.50 (79.40–93.40)	85.40 (78.50–92.10)	89.80 (82.60–96.80)	< 0.001
Left HGS/weight ratio	0.43 (0.34–0.52)	0.44 (0.36–0.53)	0.38 (0.30–0.46)	< 0.001
Left HGS(kg)	24.50 (19.25–30.50)	25.10 (20.00–31.50)	22.50 (17.25–27.25)	< 0.001
Right HGS/weight ratio	0.45 (0.36–0.55)	0.47 (0.38–0.57)	0.40 (0.32–0.48)	< 0.001
Right HGS(kg)	26.00 (20.50–32.20)	26.75 (21.00–33.25)	23.50 (18.50–29.25)	< 0.001
Sit to stand time(s)	8.88 (7.19–11.03)	8.57 (7.00–10.65)	9.74 (7.92–12.22)	< 0.001
Walking time(s)	1.04 (0.95–1.16)	1.04 (0.95–1.16)	1.03 (0.95–1.16)	0.84
Knee height(cm)	46.80 (45.00–48.80)	46.80 (45.00–49.00)	46.60 (44.90–48.50)	< 0.001
Smoking, n (%)				< 0.001
No	9,395 (88)	6,843 (85)	2,552 (96)	
Yes	1,262 (12)	1,164 (15)	98 (3.7)	
Drinking, n (%)				< 0.001
No	8,007 (75)	5,788 (72)	2,219 (84)	
Yes	2,650 (25)	2,219 (28)	431 (16)	
WBC(× 10^9/L)	5.60 (4.66–6.70)	5.56 (4.60–6.68)	5.71 (4.80–6.80)	< 0.001
HGB(g/L)	13.20 (12.20–14.30)	13.20 (12.20–14.30)	13.20 (12.20–14.20)	0.15
HCT(%)	40.10 (37.30–43.30)	40.10 (37.30–43.40)	40.00 (37.20–43.10)	0.043
MCV(fl)	91.30 (87.40–94.90)	91.30 (87.42–95.00)	91.20 (87.40–94.80)	0.56
PLT(× 10^9/L)	206.00 (163.00–249.00)	206.00 (164.00–249.00)	203.00 (162.00–248.00)	0.13
TG	121.24 (86.73–180.53)	116.81 (84.07–176.11)	136.28 (95.58–196.46)	< 0.001
Cr(mg/dl)	0.71 (0.62–0.83)	0.71 (0.62–0.83)	0.71 (0.63–0.84)	0.035
BUN(mg/dl)	14.29 (12.04–17.37)	14.29 (11.76–17.09)	14.85 (12.61–18.21)	< 0.001
HDL(mg/dl)	50.19 (43.63–57.53)	50.58 (44.02–57.92)	49.42 (42.86–56.76)	< 0.001
LDL(mg/dl)	101.16 (83.01–119.69)	100.39 (82.63–118.92)	103.47 (84.56–122.01)	< 0.001
CHO(mg/dl)	183.40 (161.39–207.72)	182.24 (160.23–206.18)	187.64 (166.02–211.97)	< 0.001
Glu(mg/dl)	95.50 (88.29–106.31)	95.50 (88.29–104.50)	97.30 (90.09–111.71)	< 0.001
UA(mg/dl)	4.50 (3.80–5.50)	4.50 (3.70–5.40)	4.70 (4.00–5.70)	< 0.001
Cys C (mg/l)	0.80 (0.69–0.92)	0.77 (0.67–0.89)	0.86 (0.75–0.98)	< 0.001
CRP(mg/l)	1.40 (0.70–2.60)	1.30 (0.70–2.40)	1.70 (0.90–3.10)	< 0.001
HbAlc(%)	5.80 (5.50–6.10)	5.70 (5.50–6.00)	5.90 (5.60–6.30)	< 0.001

### Correlation analysis among various factors

**Fig. 2:**
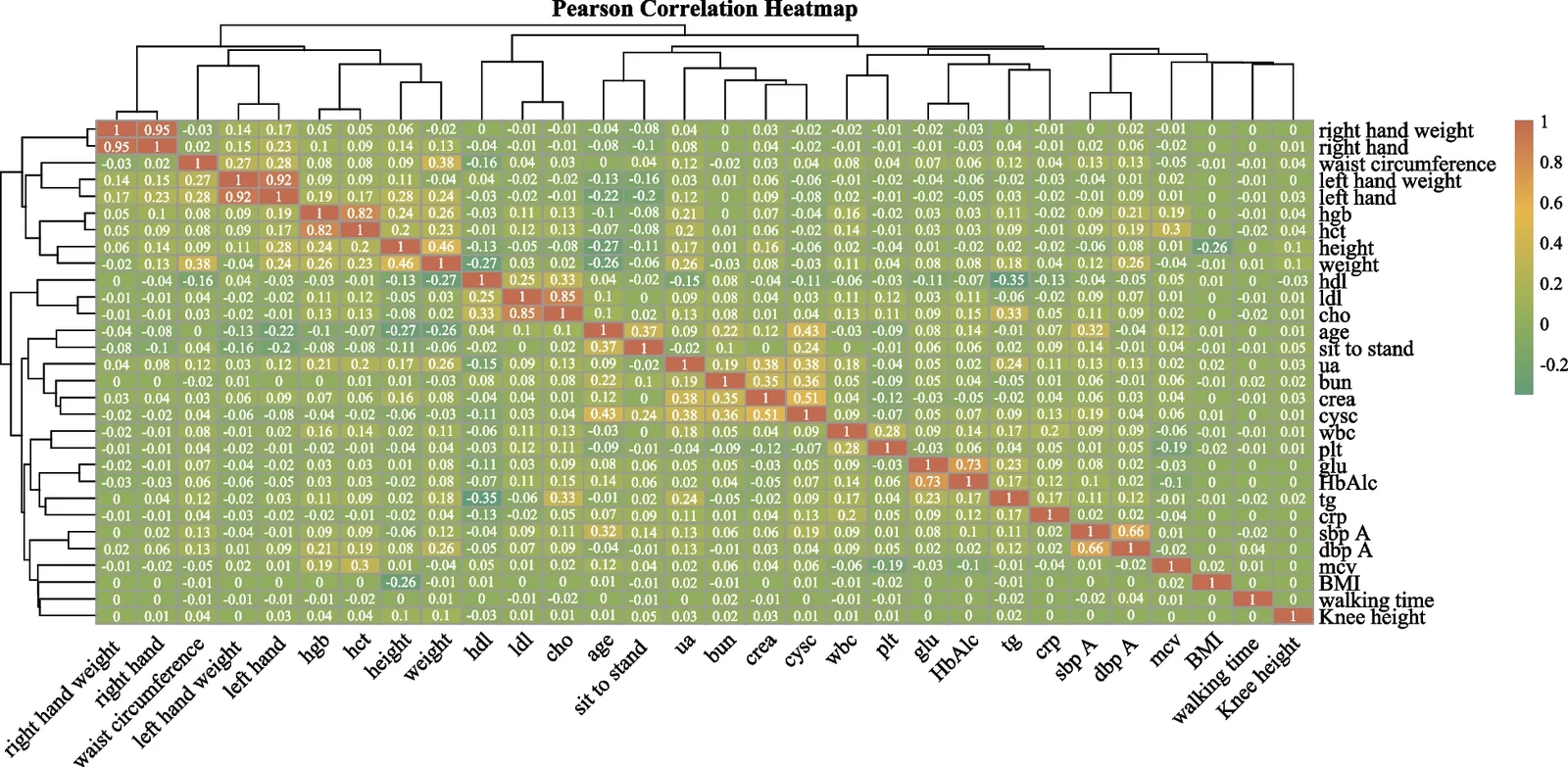
Correlation analysis diagram of characteristic. Pearson correlation analysis was used to evaluate linear correlations among continuous variables. The color gradient from green to blue indicates increasing absolute correlation coefficients and stronger negative correlations between variables, whereas the gradient from green to yellow and brown represents larger coefficients and stronger positive correlations

Pearson correlation analysis was performed on 30 continuous variables covering general demographic characteristics, cardiovascular risk factors, and frailty risk factors. Among 465 paired comparisons, significant correlations were found in 340 pairs (P < 0.05). Strong correlations were detected between systolic blood pressure and diastolic blood pressure, fasting glucose and HbA1c, total cholesterol and low-density lipoprotein cholesterol, as well as grip strength and grip strength-to-weight index. Moderate correlations existed between age and systolic blood pressure and between age and five-time sit-to-stand time, indicating interactions between frailty risk factors and cardiovascular risk factors. Binary variables were excluded from this correlation analysis and were assessed using logistic regression in subsequent analyses. Highly collinear variable pairs identified by correlation analysis provided a statistical basis for subsequent multiple regression analysis and prediction-model construction (Supplementary Table 1, Fig. 2).

### Screen variables using LASSO, simple and multiple logistic regression

**Fig. 3:**
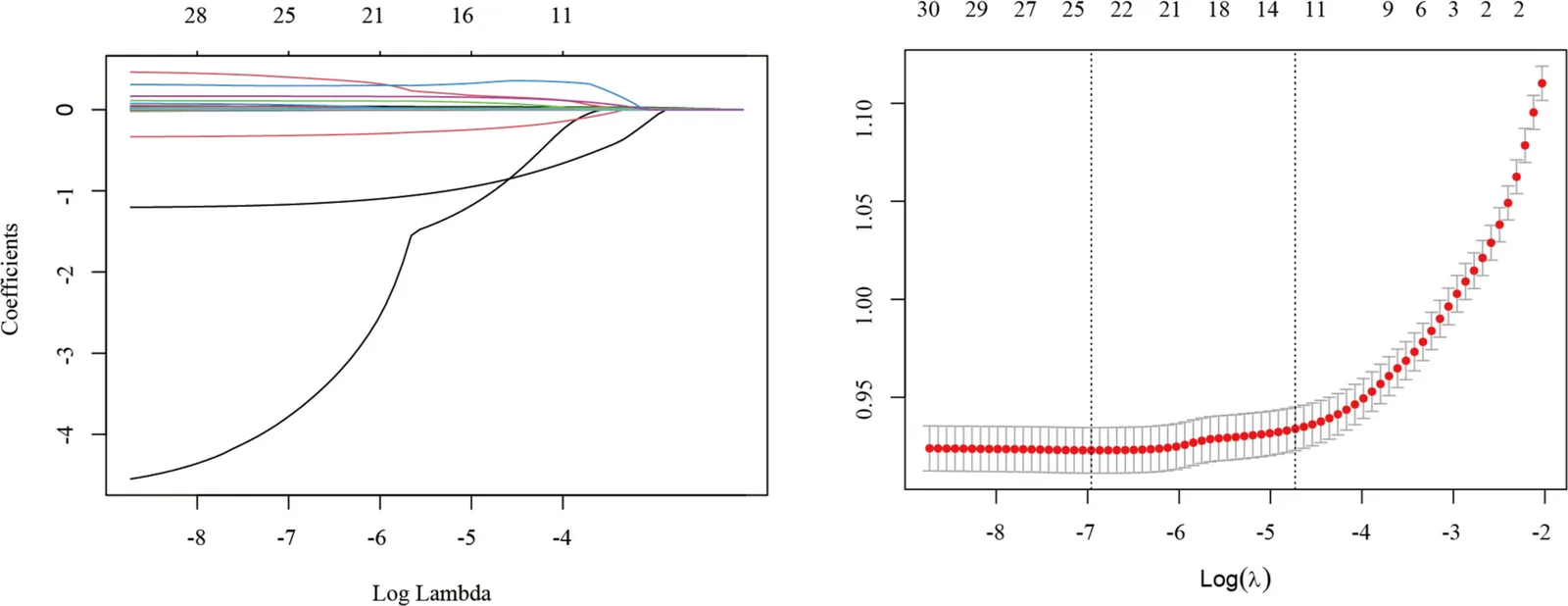
Variables screening by LASSO. For the left panel, the x-axis represents the logarithm of the penalty coefficient λ (logλ), and the y-axis represents the regression coefficients of each variable. Each colored curve corresponds to one independent variable included in this study. As logλ decreases (i.e., the penalty strength increases), the regression coefficients of all variables are gradually shrunk to 0. Finally, only the independent variables with non-zero regression coefficients are selected for inclusion. For the right panel, the x-axis is the logarithm of λ (logλ), and the y-axis is the binomial deviance. The upper and lower gray dashed lines denote the range of 1 standard error of the mean. The left vertical dashed line corresponds to λ .min, the λ value with the minimum binomial deviance, where the penalty strength is the weakest and the model retains the largest number of variables. The right vertical dashed line corresponds to λ .1se, the λ value determined by the 1-standard error rule, where the penalty strength is higher and fewer variables are retained. This study selected λ .min to determine the final set of feature variables, so as to preserve more potential indicators related to cardiovascular diseases (CVDs) and meet the needs of subsequent exploratory analyses

The LASSO was employed for feature selection. λ.min was selected as the optimal λ value through tenfold cross-validation. Eventually, 27 characteristic variables related to the occurrence of CVDs were screened out. These 27 variables were included in simple and then multiple logistic regression analyses. Finally, 13 variables were selected, namely age, gender, SBP, DBP, left HGS/weight ratio, left HGS, smoking, drinking, PLT, BUN, UA, Cys C, and HbA1c (Fig. 3, Tables 2 and 3).

**Table 2:** Univariate logistic regression of different variables

Variables	*β*	Wald	OR	Lower limit of CI	Upper limit of CI	*P*
Age	0.0613	27.1633	1.0632	1.0585	1.0679	< 0.001
Gender	0.6463	10.6582	1.9085	1.6946	2.1493	< 0.001
SBP	0.0359	28.8938	1.0366	1.0341	1.0391	< 0.001
DBP	0.0348	17.2369	1.0355	1.0314	1.0396	< 0.001
Waist circumference	0.0177	8.9766	1.0178	1.0139	1.0218	< 0.001
Left HGS/weight ratio	−4.1032	21.5711	0.0165	0.0114	0.0240	< 0.001
Left HGS	−0.0456	16.2211	0.9554	0.9502	0.9607	< 0.001
Right HGS	−0.0074	−3.1459	0.9926	0.9880	0.9972	0.002
Sit to stand time	0.0816	13.8605	1.0851	1.0726	1.0977	< 0.001
Walking time	−0.0017	−0.2389	0.9983	0.9847	1.0122	0.811
Knee height	0.0011	1.2185	1.0011	0.9993	1.0030	0.223
Smoking	−1.4930	13.4462	0.2247	0.1808	0.2793	< 0.001
Drinking	−0.6867	11.4308	0.5032	0.4473	0.5661	< 0.001
HGB	−0.0153	−1.2250	0.9849	0.9611	1.0092	0.221
HCT	−0.0077	−1.8094	0.9923	0.9841	1.0006	0.0704
MCV	0.0020	0.6510	1.0020	0.9960	1.0080	0.515
PLT	−0.0007	−2.0827	0.9993	0.9987	1.0000	0.0373
BUN	0.0403	8.0930	1.0412	1.0310	1.0514	< 0.001
HDL	−0.0103	−4.7411	0.9898	0.9856	0.9940	< 0.001
LDL	0.0029	3.7190	1.0030	1.0014	1.0045	< 0.001
UA	0.1471	8.7937	1.1584	1.1211	1.1970	< 0.001
Cys C	1.9980	17.4290	7.3746	5.8905	9.2324	< 0.001
CRP	0.0219	5.3474	1.0221	1.0140	1.0304	< 0.001
HbAlc	0.2842	12.8566	1.3286	1.2723	1.3875	< 0.001

**Table 3:** Multivariate logistic regression of different variables

Characteristic	*β*	Wald	OR	Lower limit of CI	Upper limit of CI	*P*
Age	0.0450	13.4153	1.0460	1.0391	1.0529	< 0.001
Gender	0.4396	4.6872	1.5522	1.2915	1.8654	< 0.001
SBP	0.0216	11.6870	1.0219	1.0182	1.0256	< 0.001
DBP	0.0150	4.7316	1.0151	1.0088	1.0214	< 0.001
Waist circumference	0.0007	0.5048	1.0007	0.9980	1.0033	0.613675
Left HGS/weight ratio	−4.7733	10.9762	0.0085	0.0036	0.0198	< 0.001
Left HGS	0.0529	7.2859	1.0544	1.0395	1.0695	< 0.001
Right HGS	0.0002	0.2729	1.0002	0.9989	1.0014	0.785
Sit to stand time	−0.0039	−0.5496	0.9961	0.9825	1.0100	0.583
Smoking	−1.2020	−9.2435	0.3006	0.2330	0.3879	< 0.001
Drinking	−0.3339	−4.6638	0.7161	0.6224	0.8240	< 0.001
PLT	−0.0008	−2.2292	0.9992	0.9985	0.9999	0.0258
BUN	0.0157	2.5312	1.0158	1.0035	1.0282	0.0114
HDL	−0.0009	−0.3330	0.9991	0.9939	1.0043	0.739
LDL	−0.0018	−1.9080	0.9982	0.9963	1.0000	0.0564
UA	0.1061	4.6661	1.1120	1.0635	1.1627	< 0.001
Cys C	0.2921	2.1602	1.3392	1.0274	1.7456	< 0.001
CRP	0.0077	1.5727	1.0077	0.9981	1.0175	0.1158
HbAlc	0.1666	6.9290	1.1812	1.1269	1.2382	< 0.001

### Models construction and validation based on machine learning

In this study, the 13 indicators that have been screened and determined to be related to the occurrence of CVDs as mentioned above were incorporated into six machine learning (ML) models, namely Logistic Regression (LR), eXtreme Gradient Boosting (XGBoost), Light Gradient Boosting Machine (LightGBM), Support Vector Machine (SVM), Random Forest (RF), and Decision Tree (DT). The aim is to construct and test these six ML models to make them become a clinical decision support system for assessing the risk of CVDs occurrence.

In the model development stage, 60% of the clinical characteristic data of all patients were randomly selected for model training, and the remaining 40% of the patient data were randomly and equally divided into two parts, which were used as the testing set and the validation set respectively for the internal validation of the model.

In the testing set and the validation set, the performance indicators of the six ML models were calculated. These indicators included precision rate, accuracy rate, sensitivity, specificity, F1 score, Brier score, and the area under the receiver operating characteristic curve (AUC). At the same time, by drawing the confusion matrix, the performance of the models was evaluated more comprehensively from multiple dimensions.

**Fig. 4:**
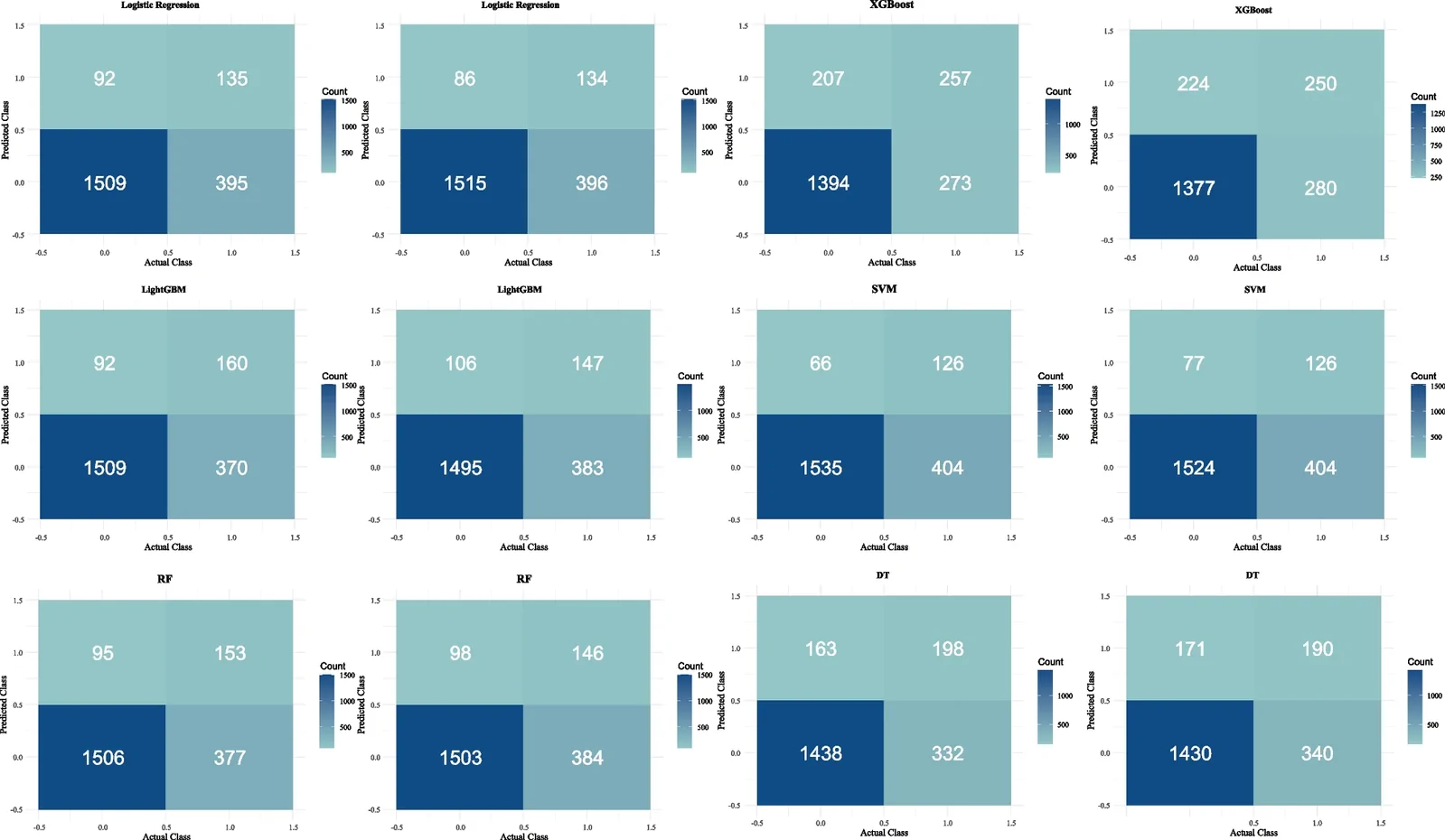
The confusion matrix of the machine learning models. X-axis: actual clinical diagnosis (CVDs-positive, CVDs-negative, left to right). Y-axis: model-predicted category (CVDs-positive, CVDs-negative, bottom to top). Matrix values = sample counts per category combination.Six machine learning models are included, each with validation-set (left) and test-set confusion matrices

**Fig. 5:**
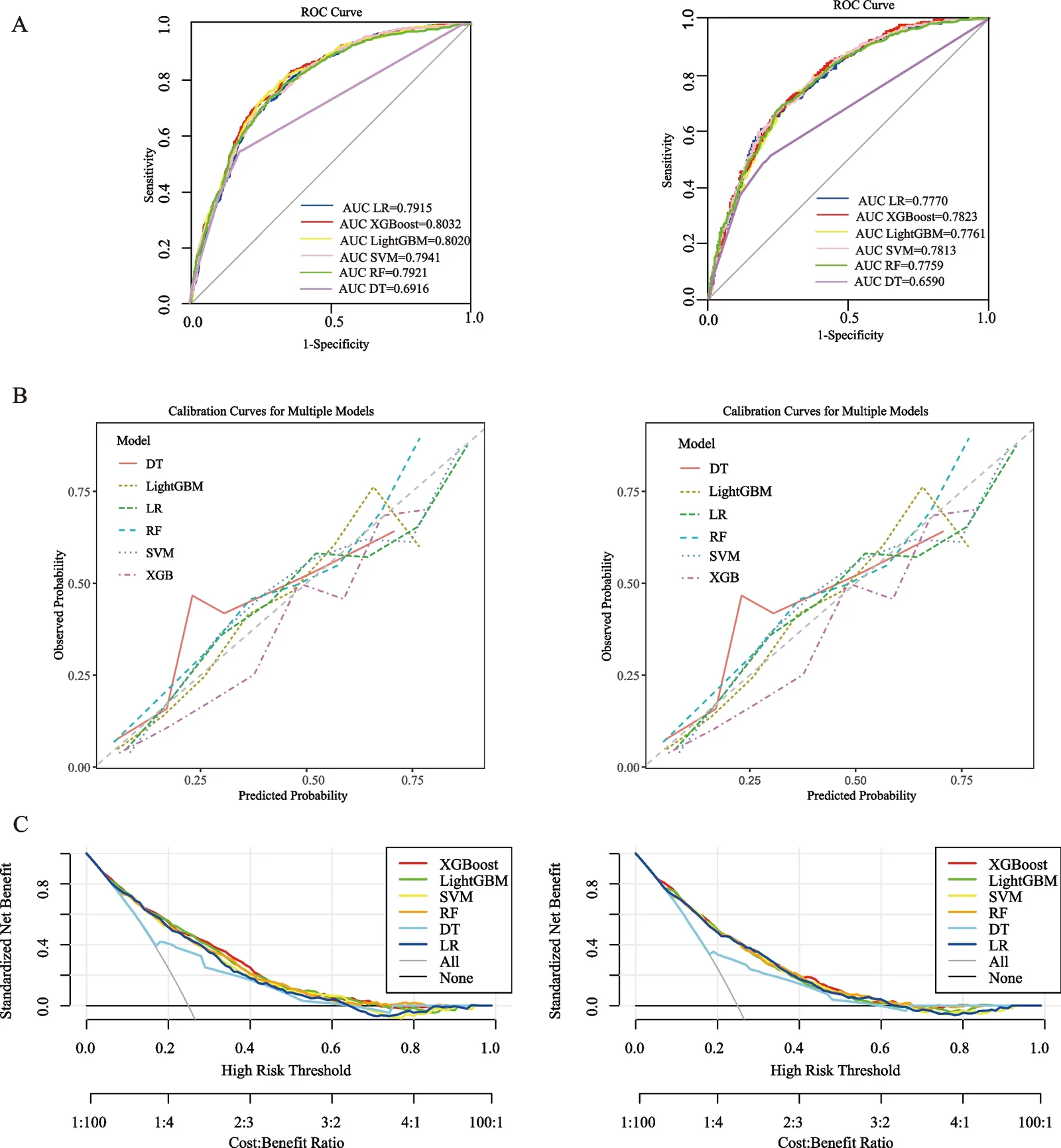
Receiver operating characteristic (ROC) curves, calibration curves, and decision curve analysis (DCA) of the six machine-learning models. In the ROC curves, the y-axis represents sensitivity and the x-axis represents 1-specificity. From top to bottom are ROC, calibration, and DCA curves, with the left column representing the validation set and the right column representing the testing set

To further evaluate the discrimination, calibration, and clinical utility of the six models in the testing set and the validation set, receiver operating characteristic curves (ROC), calibration curves, and clinical decision curves (DCA) were respectively plotted. After a comprehensive evaluation, among these six models, the XGBoost model demonstrated the best performance (Table 4, Figs. 4 and 5).

**Table 4:** Performance comparison of machine learning models

Models		Precision rate(%)	Accuracy rate(%)	Sensitivity(%)	Specificity(%)	F1 score(%)	Brier score	AUC	95%CI
	LR	78.98	77.01	94.57	23.96	86.07	0.1540	0.7770	0.75515–0.7988
	XGBoost	83.10	76.35	86.01	47.17	84.53	0.1562	0.7823	0.7611–0.8035
Test set	LightGBM	79.61	77.05	93.38	27.74	85.94	0.1541	0.7761	0.7547–0.7975
	SVM	79.05	77.43	95.19	23.77	86.37	0.1534	0.7813	0.7600–0.8028
	RF	79.65	77.38	93.88	27.55	86.18	0.1544	0.7759	0.7540–0.7978
	DT	80.79	76.02	89.32	35.85	84.84	0.1700	0.6590	0.6339–0.6840
	LR	78.94	77.38	95.32	23.21	86.36	0.1514	0.7915	0.7707–0.8122
	XGBoost	83.62	77.48	87.07	48.49	85.31	0.1497	0.8032	0.7827–0.8237
Validation set	LightGBM	80.31	78.32	94.25	30.19	86.72	0.1465	0.8020	0.7813–0.8226
	SVM	79.16	77.94	95.88	23.77	86.72	0.1505	0.7941	0.7733–0.8149
	RF	80.00	77.85	94.07	28.87	86.45	0.1491	0.7921	0.7706–0.8136
	DT	81.24	76.77	89.82	37.36	85.32	0.1637	0.6916	0.6676–0.7156

Specifically on the validation set, XGBoost achieved favorable discriminative performance with precision, accuracy, sensitivity, specificity, F1-score, Brier score and AUC of 83.62%, 77.48%, 87.07%, 48.49%, 85.31%, 0.1497 and 0.8032, respectively. DCA was performed to further evaluate the clinical utility of these predictive models. All six models yielded obvious net clinical benefit across both the training and validation datasets, with XGBoost demonstrating superior net benefit. Calibration curves were plotted in the two datasets to quantify the discrepancy between predicted and observed risks. The XGBoost model presented satisfactory calibration, indicating strong consistency between predicted and actual outcomes, which was consistent with its low Brier score.

**Fig. 6:**
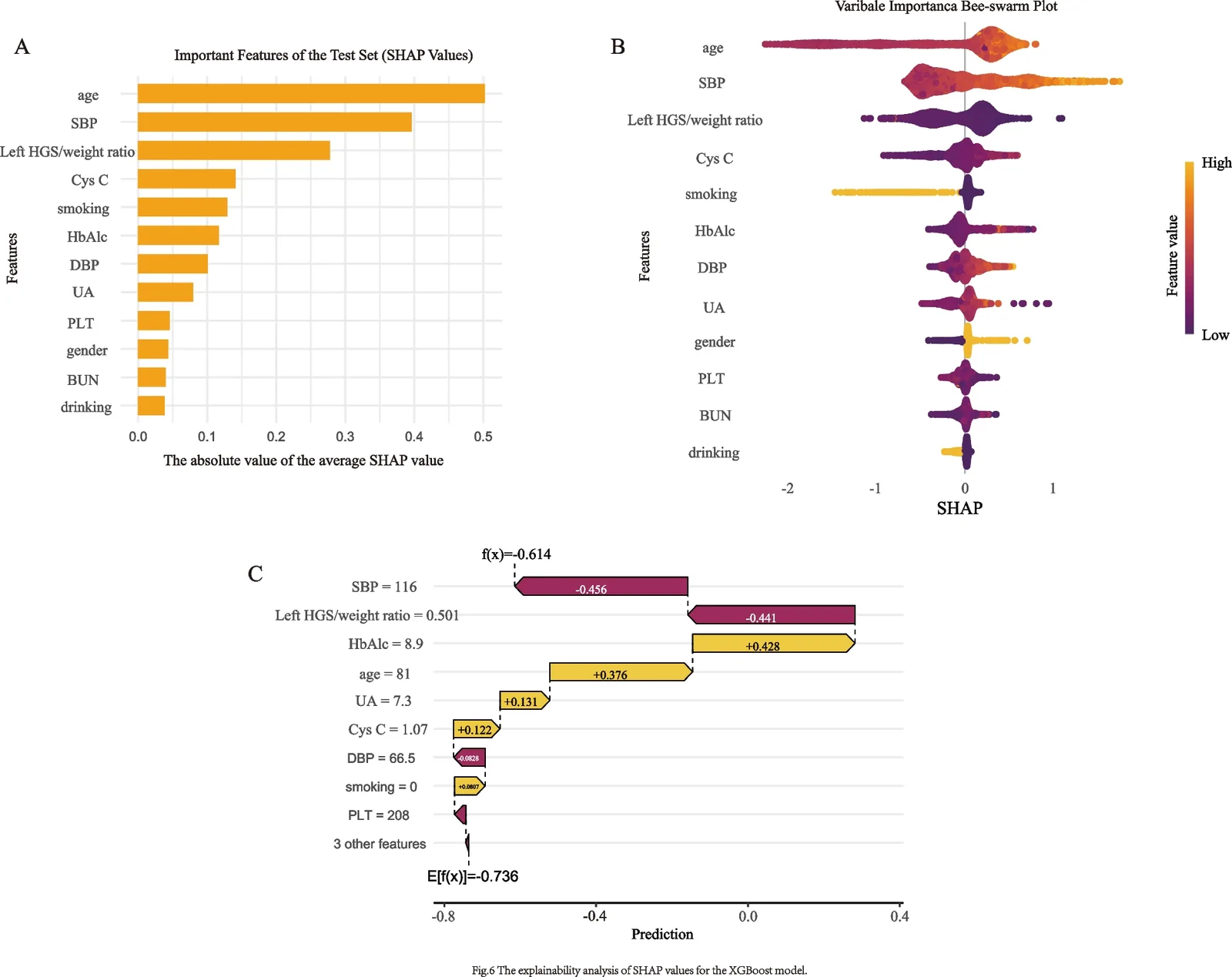
Explainability analysis of SHAP values for the XGBoost model. **A** Feature importance ranking plot. The x-axis represents the mean absolute SHAP value, and the y-axis lists model features in descending order of importance. **B** SHAP bee-swarm plot. The x-axis represents SHAP values, with positive values indicating increased predicted risk and negative values indicating decreased predicted risk; each dot represents one sample, and color depth represents feature value. **C** SHAP waterfall plot for a typical sample, showing how individual feature contributions shift the prediction from the baseline value to the final predicted risk

To interpret the XGBoost-based CVD risk model, SHAP values were calculated for each feature. In the feature-importance plot, variables were ranked by their mean absolute SHAP values. Age, SBP, and left HGS/weight ratio contributed the most to model prediction, indicating that both conventional cardiovascular factors and frailty risk factors influenced model output (Fig. 6A).

The SHAP bee-swarm and waterfall plots further illustrated the direction and magnitude of feature effects at the population and individual levels. Higher age, HbA1c, UA, and Cys C tended to increase the predicted CVD risk, whereas higher left HGS/weight ratio and lower blood pressure values tended to decrease the predicted risk (Fig. 6B–C). These results suggest that frailty risk factors, particularly HGS/weight ratio and Cys C, may add interpretive value to conventional CVD risk assessment.

## Discussion

This study aimed to identify CVD-associated factors in the Chinese middle-aged and elderly population using CHARLS. Rather than diagnosing frailty, this study focused on frailty risk factors available in CHARLS, particularly HGS-derived indicators and Cys C. The results showed that age, HbA1c, UA and Cys C were associated with higher CVD risk, whereas a higher left HGS/weight ratio and lower blood pressure values were associated with lower predicted risk in the machine-learning model.

Frailty is a complex clinical state highly associated with age, characterized by the decline of physiological functions in multiple organ systems, which significantly increases an individual's susceptibility to stressors. In clinical practice, it is crucial to screen for frailty, identify cases, and implement effective management. However, due to the current lack of sufficient evidence to support individuals and the healthcare system in carrying out effective interventions for frailty, this process faces numerous challenges [18].

The results of many existing studies have shown that frailty is closely related to a variety of adverse health outcomes and also plays a key role in the formation of the disease burden. For example, a study on the elderly population aged 75 and above in Israel found that frailty is prevalent among community-dwelling elderly people aged 75 and above in Israel, and it is associated with long-term mortality [35].

There is a two-way association between frailty and CVDs. These two conditions share several common risk factors and potential biological mechanisms. In addition, the presence of the frailty state has a significant impact on the primary and secondary prevention strategies of CVDs in adults. Meanwhile, it also serves as an obstacle to including such patients in key clinical trials and implementing advanced cardiac interventions [36].

The results of a longitudinal study on aging from India show that the association between frailty and the cardiovascular disease risk score is closer among women and the elderly population aged over 60. Thus, it is proposed that frailty screening should be adopted as a method for the primary prevention of cardiovascular diseases [37]. Recently, an expert consensus document on the management of frailty in patients with arrhythmia was released. This document provides evidence-based recommendations for the management of arrhythmia in patients with the frailty syndrome, and clarifies the current knowledge gaps and future research directions, which further highlights the important position of frailty in future health management [38].

Aging is defined as a process of gradually imbalanced accumulation of random damage and the repair mechanisms for it, eventually leading to frailty. Inflammation, like the NF-κB pathway, is crucial in this process. Moreover, factors such as genomic instability and epigenetic changes contribute to frailty [39–41].

The link between aging and CVDs is evident. Aging impacts myocardial function, causes arteriosclerosis and endothelial deterioration, raising the risk of cardiovascular diseases significantly. Aging and frailty are, to some degree, interdependent and parallel. A biobank-based study shows that lipid metabolism and DNA methylation changes, two aging hallmarks, mediate the relationship among circulating metabolites, atherosclerotic CVDs and frailty [42].

Although the role of gender was not specifically reflected in this study due to limitations in data and models, previous studies have shown that there are gender differences in both CVDs and frailty. Overall, women face higher risks in cardiovascular diseases and frailty than men. Due to menstrual cycle changes and pregnancy, in addition to traditional risk factors, women also encounter special factors like menarche and menopause age, polycystic ovary syndrome, infertility, and assisted reproductive technology application, which increase their CVDs risk and burden. As women age, the decline in estrogen levels causes muscle weakness and other conditions, and 17β-estradiol is crucial for women's health. Owing to the differences in sex hormone patterns between genders, postmenopausal women have a higher frailty risk than men [43, 44].

HGS, an indicator of muscle strength, effectively gauges general health, notably predicting early all–cause and cardiovascular mortality, and disability [45]. WHO advises adults to do muscle–strengthening for major muscle groups at moderate or higher intensity for at least 2 days/week (3 days/week for older adults) besides aerobic exercise [46].

HGS varies by gender and age. Defining normal HGS is key for evaluation. A review of 2.4 million adults from 69 regions shows HGS peaks at 30–39 (49.7 kg for men, 29.7 kg for women), then declines. The decline speeds up from mid–to late adulthood, with men having a slightly larger drop in mid–adulthood [47].

As an endogenous marker of renal function, Cys C can estimate the glomerular filtration rate and reflect pathophysiological processes beyond renal function [48]. Studies show that elevated Cys C levels are associated with inflammation, oxidative stress, and endothelial dysfunction, which play key roles in the development of kidney diseases and various CVDs [49]. A study based on the NHANES database in the US reveals that patients with metabolic syndrome and high Cys C levels have a higher risk of all-cause, cardiovascular, and cancer mortality, indicating that Cys C can predict all-cause and cardiovascular mortality in such patients [50]. Recent research suggests a link between Cys C and frailty. The difference between Cys C and the glomerular filtration rate estimated by creatinine is associated with a reduced frailty risk, which can be explained by muscle mass [51]. Additionally, a study on community-dwelling elderly people shows that a low Cr/Cys C ratio is correlated with an increased frailty incidence in elderly men, but this relationship is not found in elderly women [52].

The study findings have the following implications for health policies and clinical screening: first, identifying modifiable and non-modifiable risk factors helps policymakers allocate resources strategically for targeted CVD prevention, enhancing population cardiovascular health and reducing disease burden. Second, clinical screening may expand beyond traditional metrics (e.g., blood pressure, lipids, and glucose) to include HbA1c, UA, Cys C, and simple physical-performance indicators for comprehensive risk assessment. High-risk groups such as smokers, drinkers, and older adults require intensified monitoring. Third, promoting grip strength training and weight management—especially among older adults—is clinically relevant, because the HGS/weight ratio is not a diagnostic criterion for frailty but is a simple functional indicator related to physical reserve and frailty risk.

However, limitations exist. This study only used cross-sectional data from the 2015 CHARLS and lacked follow-up information, making it difficult to clarify the progression of CVDs and dynamic changes in relevant risk factors. In addition, as a long period has elapsed, residents’ lifestyles, CVDs prevention strategies and composition of risk factors have changed, restricting the direct extrapolation of our findings. Moreover, prevalent CVDs were defined using self-reported physician diagnosis and included hypertension, heart disease and stroke; therefore, misclassification and overlap between the outcome definition and blood-pressure-related predictors cannot be fully excluded. Further research may incorporate the 2018 and 2020 CHARLS datasets to conduct temporal external validation and model updating, so as to address limitations related to timeliness and insufficient longitudinal data. Some disease information relied on self-reports, with uncertain reliability. Privacy concerns might cause data concealment, and recall of past health details could introduce bias. Also, variables were selected based on prior research and clinical experience, potentially missing other influencing factors and creating bias.

## Conclusion

Based on data analysis and the XGBoost-based machine-learning model, age, HbA1c, UA and Cys C were associated with increased CVD risk in the Chinese middle-aged and elderly population, while higher left HGS/weight ratio and lower blood pressure values were associated with lower predicted risk. These variables should be interpreted as frailty risk factors or CVD-related indicators rather than as a complete frailty diagnosis. Strengthening frailty-risk screening, physical performance assessment, and individualized risk management may contribute to CVD prevention.

## Supplementary Information

Supplementary Material 1.

## Data Availability

The datasets generated during and/or analyzed during the current study are available from the CHARLS dataset (https://charls.pku.edu.cn/).
